# Hospital and regional variations in intensive care unit admission for patients with invasive mechanical ventilation

**DOI:** 10.1186/s40560-024-00736-0

**Published:** 2024-06-05

**Authors:** Hiroyuki Ohbe, Nobuaki Shime, Hayato Yamana, Tadahiro Goto, Yusuke Sasabuchi, Daisuke Kudo, Hiroki Matsui, Hideo Yasunaga, Shigeki Kushimoto

**Affiliations:** 1 Department of Emergency and Critical Care Medicine, Tohoku University Hospital, 1-1 Seiryo-Machi, Aoba-Ku, Sendai, 980-8574 Japan; 2 Department of Clinical Epidemiology and Health Economics, School of Public Health, The University of Tokyo, 7-3-1 Hongo, Bunkyo-Ku, Tokyo, 113-0033 Japan; 3 Department of Emergency and Critical Care Medicine, Graduate School of Biomedical and Health Sciences, Hiroshima University, 1-2-3 Kasumi, Minami-Ku, Hiroshima, 734-8551 Japan; 4 Data Science Center, Jichi Medical University, 3311-1 Yakushiji, Shimotsuke, Tochigi 329-0498 Japan; 5 TXP Medical Co., Ltd., 41-1 H1O Kanda 706, Kanda Higashimatsushita-Cho, Chiyoda-Ku, Tokyo, 101-0042 Japan; 6 Department of Real-World Evidence, Graduate School of Medicine, The University of Tokyo, 7-3-1 Hongo, Bunkyo-Ku, Tokyo, 113-0033 Japan; 7 Division of Emergency and Critical Care Medicine, Tohoku University Graduate School of Medicine, 2-1 Seiryo-Machi, Aoba-Ku, Sendai, Miyagi 980-8575 Japan; 8 Department of Health Services Research, Graduate School of Medicine, The University of Tokyo, 7-3-1 Hongo, Bunkyo-Ku, Tokyo, 113-0033 Japan

**Keywords:** Intensive care unit admission, Invasive mechanical ventilation, Health service research, Critical care delivery, Multilevel analysis

## Abstract

**Background:**

Patients who receive invasive mechanical ventilation (IMV) in the intensive care unit (ICU) have exhibited lower in-hospital mortality rates than those who are treated outside. However, the patient-, hospital-, and regional factors influencing the ICU admission of patients with IMV have not been quantitatively examined.

**Methods:**

This retrospective cohort study used data from the nationwide Japanese inpatient administrative database and medical facility statistics. We included patients aged ≥ 15 years who underwent IMV between April 2018 and March 2019. The primary outcome was ICU admission on the day of IMV initiation. Multilevel logistic regression analyses incorporating patient-, hospital-, or regional-level variables were used to assess cluster effects by calculating the intraclass correlation coefficient (ICC), median odds ratio (MOR), and proportional change in variance (PCV).

**Results:**

Among 83,346 eligible patients from 546 hospitals across 140 areas, 40.4% were treated in ICUs on their IMV start day. ICU admission rates varied widely between hospitals (median 0.7%, interquartile range 0–44.5%) and regions (median 28.7%, interquartile range 0.9–46.2%). Multilevel analyses revealed significant effects of hospital cluster (ICC 82.2% and MOR 41.4) and regional cluster (ICC 67.3% and MOR 12.0). Including patient-level variables did not change these ICCs and MORs, with a PCV of 2.3% and − 1.0%, respectively. Further adjustment for hospital- and regional-level variables decreased the ICC and MOR, with a PCV of 95.2% and 85.6%, respectively. Among the hospital- and regional-level variables, hospitals with ICU beds and regions with ICU beds had a statistically significant and strong association with ICU admission.

**Conclusions:**

Our results revealed that primarily hospital and regional factors, rather than patient-related ones, opposed ICU admissions for patients with IMV. This has important implications for healthcare policymakers planning interventions for optimal ICU resource allocation.

**Supplementary Information:**

The online version contains supplementary material available at 10.1186/s40560-024-00736-0.

## Background

Guidelines for intensive care unit (ICU) admission recommend that patients receiving invasive mechanical ventilation (IMV) be treated in the ICU rather than outside the ICU [1, 2]. This recommendation is supported by previous evidence showing lower in-hospital mortality rates in patients who received IMV in the ICU compared to those who were treated outside of it [3, 4].

Nevertheless, it is evident that a considerable number of patients on IMV are treated outside ICUs [5–7]. Both single- and multi-centre studies have shown that patient-level variables—such as age, comorbidity score, presence of malignancies, surgical history, and severity score—are associated with ICU admission [5–9]. One questionnaire-based study found that ICU bed availability, patient and family preferences, and ethical variations were also associated with ICU admission in this patient group. [10]

The decision to admit patients to the ICU involves a complex interplay of different factors, including patient characteristics, institutional resources, and regional healthcare structures. However, a considerable knowledge gap exists regarding the factors influencing ICU admissions and the extent of variability across different healthcare institutions and regions. To the best of our knowledge, no studies have yet quantitatively examined which patient-, hospital-, and region-specific factors influence the ICU admission of patients on IMV.

Therefore, this study aimed to investigate hospital and regional variations in the ICU admission of patients receiving IMV, using multilevel analyses. Understanding the factors influencing ICU admissions can help visualise variations across hospitals and regions, as well as provide valuable insights for healthcare resource allocation and quality improvement initiatives.

## Methods

### Study design and data collection

This retrospective observational study used data from a nationwide inpatient administrative database, as well as medical facility statistics, from Japan. We used the Japanese Diagnosis Procedure Combination inpatient (DPC) database, which contains discharge abstracts and administrative claims data from > 1200 acute-care hospitals in Japan that voluntarily contributed to the database [11]. The database includes the following patient-level data for all hospitalisations: demographics; diagnoses (recorded using International Classification of Diseases, Tenth Revision [ICD-10] codes); daily procedures (recorded using Japanese medical procedure codes); daily drug administrations; and admission and discharge statuses. A previous validation study of the DPC database showed that both the sensitivity and specificity of the procedures were high (> 90%) [12] .

We also used facility information and statistical data from the Survey of Medical Institutions 2018 [13], which was provided by the Ministry of Health, Labour and Welfare of Japan; and included medical facility statistics for all Japanese hospitals as of 1 July, 2018. The Survey of Medical Institutions included information on secondary medical areas, hospital zip codes, ward types, number of hospital beds in each ward, and hospital type. Secondary medical areas in Japan comprise 339 jurisdictions. Regional healthcare systems are planned based on each secondary medical area, to maintain general inpatient medical care, including IMV.

### Study population

Using DPC data from 1 April, 2018, to 31 March, 2019 we identified all patients aged ≥ 15 years who received IMV during their hospitalisations. The choice of this period was informed by the need to evaluate ICU admissions in a non-disaster setting prior to the coronavirus disease 2019 pandemic. IMV was identified based on the Japanese procedure code J045, which includes IMV during hospitalisation but not during general anaesthesia, cardiopulmonary resuscitation, or at-home mechanical ventilation. The day of IMV initiation was defined as the earliest date of receiving IMV during hospitalization. If a patient received multiple courses of IMV during the same hospitalisation period, only the first course was included. Patients who received IMV only in rehabilitation or chronic beds (nurse-to-patient ratios of 1:13 and 1:15, respectively) were excluded because they were considered ventilator-dependent. Because hospital contributions to the DPC database were voluntary, we excluded patients on IMV who were admitted to hospitals in secondary medical areas—where the DPC database covers < 80% of all acute care beds (nurse-to-patient ratio of 1:10 or higher).

### Definition of critical care beds

ICU was defined as a separate unit providing critical care services with at least one physician on site 24 h per day, at least two board-certified intensivists working full-time (only required for recourse-rich ICUs), around-the-clock nursing, the equipment necessary to care for critically ill patients, and a nurse-to-patient ratio of 1:2 [1, 14]. The high-dependency care unit (HDU), also called an ‘intermediate care unit’ or ‘step down unit’, was considered a unit where critical care services are provided to patients whose care level needs fell between those of the ICU and the general wards [15, 16]. HDU was therefore defined as almost identical to ICU but differed from ICU in that it had a nurse-to-patient ratio of 1:4 or 1:5 and did not require board-certified intensivists [14]. A general ward was defined as a general unit in an acute care hospital, with a nurse-to-patient ratio of 1:7 or 1:10, and without the necessary equipment to care for critically ill patients. Details of the Japanese procedure codes are provided in Supplemental Table 1.

### Outcome and variables

The primary outcome was ICU admission on the day of IMV initiation. When patients were treated in hospitals without ICU beds and transferred to other hospitals on the day of IMV initiation, this was also considered an outcome because we considered them to have been transferred for ICU admission. The secondary outcomes were ICU or HDU admission after the day of IMV initiation, in-hospital mortality, length of hospital stay, length of IMV, length of ICU stay, and total hospitalisation costs.

The patient-level variables included age, sex, body mass index at admission, Charlson comorbidity index score, cognitive function before admission (no dementia, mild dementia, or moderate/severe dementia), long-term care needs before admission, home medical care before admission, location before hospitalization (home, another hospital, or nursing home), admission on a weekend (i.e. on Saturday or Sunday), ambulance use, emergency admission, surgery under general anaesthesia before IMV, cardiopulmonary resuscitation on the day of IMV initiation, length of hospital stay before IMV, primary diagnosis at admission, and geodetic distance (i.e. the length of the shortest curve between two points along the surface of a mathematical model of the earth) from the patient’s home to the nearest hospital with ICU beds.

Hospital-level variables included hospitals with ICU beds, number of ICU beds, number of HDU beds, number of acute-care beds, academic hospitals, tertiary emergency hospitals, annual number of ambulances, and annual IMV case volume per hospital.

Secondary medical areas were selected at the regional level. The regional-level variables were regions with ICU beds and the number of ICU, HDU, and acute care beds per 100,000 population in the region. Population data were obtained from the 2018 Japanese Population Census, and data for each secondary medical area were age-adjusted, sex-adjusted, and standardised to the general 2018 Japanese population [17].

### Statistical analysis

We applied the framework of multilevel analysis to estimate the effects of variables measured at the subject and cluster levels, as described by Austin et al. [18–20] The study outcome of ICU admission was analysed using multilevel logistic regression with patients at the subject level, and hospitals or regions at the cluster level. The following three models were applied separately to analyse the hospitals and regions as clusters. Model 1 included only random intercepts for the clusters—that is, we allowed the baseline risk of ICU admission to vary between clusters and quantified the amount of variation in ICU admissions between clusters. Model 2 incorporates patient-level variables and random intercepts for each cluster. Model 3 integrated patient- and cluster-level variables, along with random intercepts for each cluster. The details of the models are presented in Supplemental Table 2. Using Model 3, we plotted the posterior means of the random effects to evaluate the cluster variation.

To estimate the general contextual effects (i.e., the effect of the cluster itself on subject outcomes), we calculated the intraclass correlation coefficient (ICC), median odds ratio (MOR), and proportional change in variance (PCV). An ICC of 0% indicated no cluster effect, whereas values approaching 100% indicated that the cluster itself determined ICU admission [18]. The MOR represents the magnitude of the cluster effect on a familiar odds ratio scale (MOR = 1 indicates lack of effect; larger or smaller values indicate greater variation) [18]. The PCV explained by adding patient- and cluster-level variables was calculated as the difference between Models 1 and 2, and between Models 2 and 3. To estimate the general contextual effects differently, we also calculated the changes in the area under the receiver operating characteristic curve (AUC) between the models, with Model 1 as the reference [18].

To estimate the specific contextual effects (i.e., the effect of cluster-level variables on subject outcomes), we calculated the 80% interval odds ratio (IOR-80%) and proportion of opposed odds ratios (POOR) in Model 3 [21, 22]. IOR-80% and POOR summarise the odds ratios of random comparisons of the exposed and non-exposed clusters. IOR-80% represents the distribution of the odds ratios and POOR is the proportion of odds ratios opposite to the overall odds ratio. Whenever the margins of the IOR cross 1, the effect of the cluster-level variable is considered small relative to the amount of variation between clusters. POOR values can range from 0 to 50%, with larger ones implying that the association is more heterogeneous.

Descriptive statistics of variables are presented as medians and interquartile ranges (IQRs), means and standard deviations (SDs), or counts and percentages, as appropriate. Differences between groups were evaluated using standardised mean differences. The association between the number of ICU beds and the proportion of ICU admissions in a given hospital or secondary medical area is shown graphically using a fractional-polynomial prediction plot. All reported *p*-values are two-sided, and values of *p* < 0.05 were considered statistically significant. All analyses were performed using STATA/SE version 17.0 software (StataCorp LLC, College Station, TX, USA).

Sensitivity analyses were performed for hospitals and regions with at least one ICU bed to assess the general and specific contextual effects of hospitals and regions with ICU beds.

## Results

In the 2018 Survey of Medical Institutions, the total number of ICU beds per 100,000 population in Japan was 5.4. Of the 339 secondary medical areas in Japan, 199 were excluded because the DPC database covered < 80% of all acute-care beds. The characteristics of the excluded and included patients showed a negligible imbalance in terms of most variables (Supplemental Table 3). In the end, 83,346 patients aged ≥ 15 years who received IMV were eligible for our analysis, which covered 546 hospitals in 140 secondary medical areas (Supplemental Fig. 1). Of these, 33,642 (40.4%) were treated in ICU beds on the day of IMV initiation, and 49,704 (59.6%) were treated in HDUs or general wards. Of the 33,642 ICU patients, 108 were transferred from hospitals without ICU beds to other hospitals on the day of IMV initiation. Of the 49,704 HDU and general ward patients, 20,907 (42.1%) were treated in HDUs and 28,797 (57.9%) were treated in general wards. Among the same HDU and general ward patient group, 1,231 (2.5%) were admitted to ICU beds after the day of IMV.

The mean age of the 83,346 eligible patients was 72.1 years (standard deviation, 15.4 years), and 60.6% were male (Table 1). The most common diagnosis was acute heart failure (15.3%), followed by postcardiac arrest (12.2%), acute coronary syndrome (9.0%), and stroke (8.8%).

**Table 1:** Patient-, hospital-, and region-level characteristics of patients treated with invasive mechanical ventilation in an ICU, HDU, or ward

Variables	ICU	HDU/ward	SMD
	*N* = 33,642	*N* = 49,704	
*Patient-level*			
Age, years, mean (SD)	69.6 (14.9)	73.8 (15.5)	–28
Male, *n* (%)	20,952 (62.3)	29,518 (59.4)	6
BMI at admission, kg/m^2^, *n* (%)			
< 18.5	4535 (13.5)	9309 (18.7)	–14
18.5–24.9	18,199 (54.1)	21,523 (43.3)	22
25.0–29.9	6241 (18.6)	6279 (12.6)	16
≥ 30.0	1703 (5.1)	1985 (4.0)	5
Missing	2964 (8.8)	10,608 (21.3)	–36
CCI, mean (SD)	1.3 (1.6)	1.2 (1.6)	9
Cognitive function, *n* (%)			
No dementia	28,194 (83.8)	37,227 (74.9)	22
Mild dementia	3115 (9.3)	6,513 (13.1)	–12
Moderate/severe dementia	2333 (6.9)	5964 (12.0)	–17
Long-term care-needs, *n* (%)			
No care-needs	33,598 (99.9)	49,445 (99.5)	7
SL1-2 & CNL1-2	24 (0.1)	101 (0.2)	–4
CNL3-5	20 (0.1)	158 (0.3)	–6
Home medical care, *n* (%)	1136 (3.4)	4388 (8.8)	–23
Admission on a weekend, *n* (%)	8008 (23.8)	11,878 (23.9)	0
Location before admission, *n* (%)			
Home	29,086 (86.5)	42,056 (84.6)	5
Other hospitals	3474 (10.3)	3541 (7.1)	11
Nursing home	1082 (3.2)	4107 (8.3)	–22
Ambulance use, *n* (%)	19,161 (57.0)	31,682 (63.7)	–14
Emergency admission, *n* (%)	24,178 (71.9)	42,749 (86.0)	–35
Surgery, *n* (%)	6709 (19.9)	3154 (6.3)	41
CPR, *n* (%)	3299 (9.8)	13,211 (26.6)	–45
Length of stay before IMV, *n* (%)			
On the day of admission	12,183 (36.2)	28,720 (57.8)	–44
On the next day of admission	6549 (19.5)	5796 (11.7)	22
Day 3–6	7875 (23.4)	6483 (13.0)	27
Day 7	7035 (20.9)	8705 (17.5)	9
Primary diagnoses, *n* (%)			
Acute heart failure	3650 (10.8)	9063 (18.2)	–21
Post cardiac arrest	2625 (7.8)	7581 (15.3)	–23
Acute coronary syndrome	3018 (9.0)	4297 (8.6)	1
Stroke	4537 (13.5)	2981 (6.0)	25
Cancer	3426 (10.2)	3516 (7.1)	11
Aortic dissection or aneurysm	3887 (11.6)	1860 (3.7)	30
Abdominal diseases	2755 (8.2)	2970 (6.0)	9
Pneumonia	1072 (3.2)	4218 (8.5)	–23
Trauma	2080 (6.2)	2394 (4.8)	6
Sepsis	1766 (5.2)	1413 (2.8)	12
Aspiration	801 (2.4)	2502 (5.0)	–14
Chronic lower respiratory diseases	282 (0.8)	1577 (3.2)	–17
Home to nearest ICU, km, median (IQR)	4.5 (2.2–10.3)	5.6 (2.7–13.1)	–15
*Hospital-level*			
Hospitals with ICU beds, *n* (%)	33,534 (99.7)	29,249 (58.8)	117
ICU beds, median (IQR)	13 (8–22)	6 (0–10)	89
HDU beds, median (IQR)	18 (9–28)	12 (0–24)	26
Acute-care beds, median (IQR)	576 (427–792)	414 (250–600)	63
Academic hospital, *n* (%)	10,178 (30.3)	6,182 (12.4)	45
Tertiary emergency hospital, *n* (%)	22,289 (66.3)	23,763 (47.8)	38
Ambulance cases, median (IQR)	4476 (2944–6187)	3478 (2000–5571)	31
IMV cases, median (IQR)	401 (281–570)	260 (122–435)	49
*Regional-level*			
Regions with ICU beds, *n* (%)	33,599 (99.9)	42,736 (86.0)	56
ICU beds per million, median (IQR)	7.3 (5.0–10.1)	5.0 (2.9–7.8)	57
HDU beds per million, median (IQR)	11.9 (8.7–16.8)	11.4 (8.0–15.0)	18
Acute-care beds per million, median (IQR)	633 (523–697)	605 (474–679)	20

*ICU* intensive care unit, *HDU* high-dependency care unit, *SMD* standardised mean difference, *SD* standard deviation, *BMI* body mass index, *CCI* Charlson Comorbidity Index, *SL* support level, *CNL* care needs level, *CPR* cardiopulmonary resuscitation, *IMV* invasive mechanical ventilation, *IQR* interquartile range

The crude in-hospital mortality for patients treated in ICUs and HDUs or general wards were 24.7% and 49.0%, respectively (Supplemental Table 4).

### Hospital variation in intensive care unit admission

**Fig. 1:**
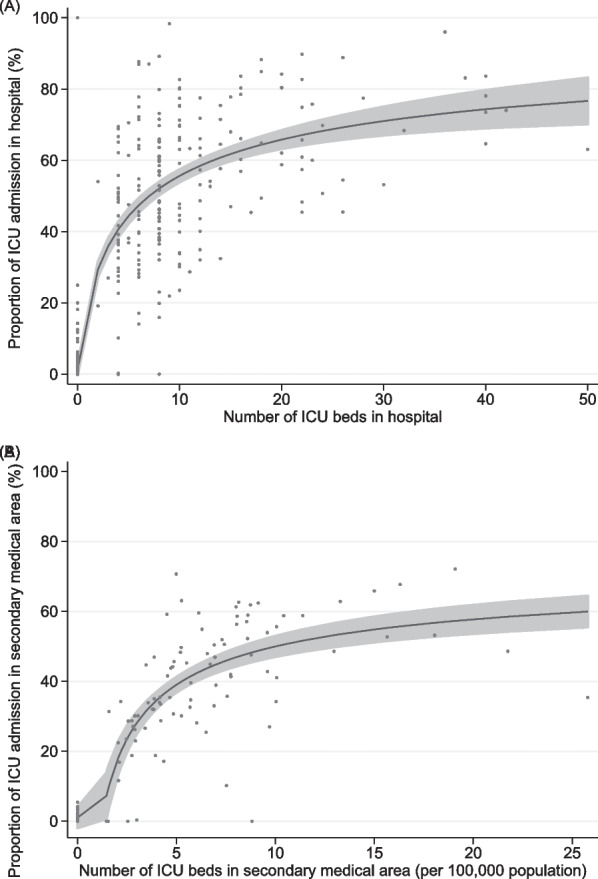
Association between (**A**) the proportion of ICU admissions and the number of ICU beds per hospital; and (**B**) the proportion of ICU admissions and the number of ICU beds in secondary medical areas, per 100,000 population, as visualised by a fractional-polynomial prediction plot with 95% confident intervals and an overlaid scatterplot. *ICU* intensive care unit

**Fig. 2:**
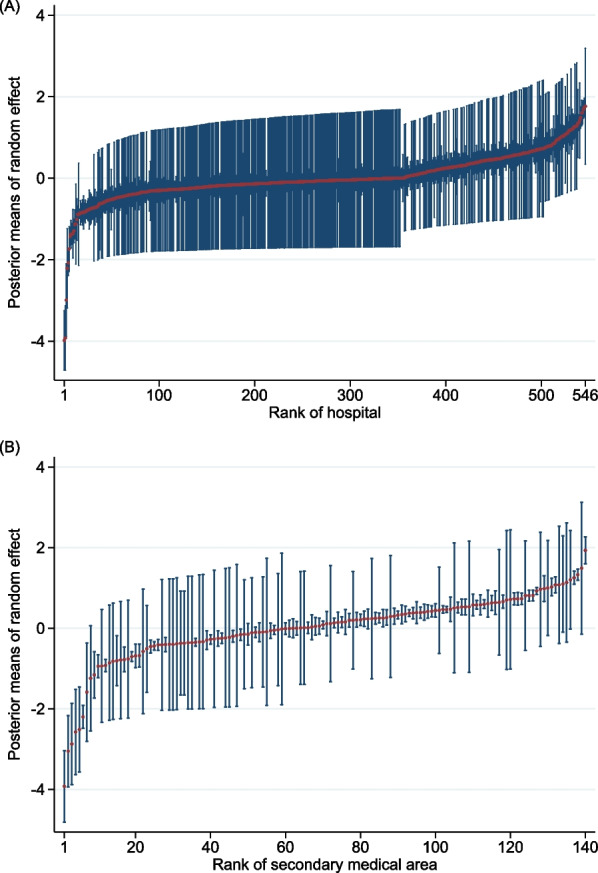
Posterior means of random effects to evaluate the cluster variations in **A** hospital and **B** secondary medical areas, using Model 3. Model 3 was a multilevel logistic regression model with patient-level covariates, cluster-level variables, and random intercepts for the clusters

The median number of ICU beds among analysed 546 hospitals was 0 (IQR 0 to 6), and 346 (63.4%) were not equipped with any ICU bed (Supplemental Table 5), which accounted for 24.7% (*n* = 20,563/83,346) of the IMV patients in those 346 hospitals. ICU admission rates varied widely between the 546 participating hospitals, from 0 to 100% (median, 0.7%; IQR, 0–44.5%). The hospital transfer rate on the day of IMV initiation in hospitals without ICU beds was 0.5% (*n* = 108/20,563). The ICU admission rate increased to ~ 60% until the number of ICU beds reached 12, after which it plateaued (Fig. 1A). The posterior means of the random effects in Model 3 showed significant differences between hospitals (Fig. 2A). In Model 1, the general contextual effect of the hospital cluster was large, with an ICC of 82.2% and an MOR of 41.4 (Table 2). Accounting for patient-level variables in Model 2 did not change the ICC or MOR, with a PCV of 2.3%. Further adjustment for hospital-level variables in Model 3 decreased the ICC and MOR, with a PCV of 95.2%. Among the hospital-level variables, hospitals with ICU beds had a statistically significant and strong association with outcomes (IOR-80%, 50.2–1135 and POOR, 0.0; Table 3). The full results of Models 2 and 3 for the multilevel analyses of hospital clusters are shown in Supplemental Table 6.

**Table 2:** General contextual effects of hospital- and regional-level variables in our multilevel logistic regression analysis

Statistic	Model 1	Model 2	Model 3
*Hospital-level*			
ICC (%)	82.2 (79.1, 85.0)	81.9 (78.7, 84.7)	18.3 (15.2, 21.9)
MOR	41.4 (25.6, 57.2)	39.7 (24.8, 54.5)	2.27 (2.06, 2.48)
PCV (%)			
Models 1 and 2	Ref	2.3	–
Models 2 and 3	–	Ref	95.2
AUC	0.837	0.891	0.891
Difference in AUCs			
Models 1 and 2	Ref	0.054	–
Models 2 and 3	–	Ref	0
*Regional-level*			
ICC (%)	67.3 (60.5, 73.5)	67.5 (60.7, 73.7)	22.2 (16.8, 28.7)
MOR	12.0 (7.55, 16.4)	12.1 (7.62, 16.6)	2.52 (2.12, 2.92)
PCV (%)			
Models 1 and 2	Ref	–1.0	–
Models 2 and 3	–	Ref	86.2
AUC	0.713	0.821	0.821
Difference in AUCs			
Models 1 and 2	Ref	0.108	–
Models 2 and 3	–	Ref	0

Model 1: multilevel logistic regression with random intercepts for clusters; Model 2: multilevel logistic regression with patient-level covariates and random intercepts for clusters; and Model 3: multilevel logistic regression with patient-level variables, cluster-level variables, and random intercepts for clusters

*ICU* intraclass correlation coefficient, *ICC* intensive care unit, *MOR* median odds ratio, *PCV* proportional change in variance, *AUC* area under the receiver operating characteristic curve

**Table 3:** Specific contextual effects of hospital- and regional-level variables in our multilevel logistic regression analysis for Model 3

Variables	Odds ratio (95% CI)	IOR-80%	POOR
*Hospital-level*			
Hospitals with ICU beds	239 (172, 332)	(50.2, 1135)	0.0
ICU beds	1.10 (1.08, 1.12)	(0.23, 5.23)	46.8
HCU beds	0.99 (0.98, 1.00)	(0.21, 4.72)	49.8
Acute-care beds	1.00 (1.00, 1.00)	(0.21, 4.75)	50.0
Academic hospital	1.13 (0.70, 1.80)	(0.24, 5.36)	46.1
Tertiary emergency hospital	0.62 (0.45, 0.84)	(0.13, 2.93)	34.5
Ambulance cases	1.00 (1.00, 1.00)	(0.21, 4.75)	50.0
IMV cases	1.00 (1.00, 1.00)	(0.21, 4.75)	50.0
*Regional-level*			
Regions with ICU beds	52.7 (29.1, 95.3)	(9.09, 305.48)	0.2
ICU beds per million	1.11 (1.06, 1.17)	(0.19, 6.46)	46.9
HDU beds per million	1.00 (0.98, 1.03)	(0.17, 5.8)	50.0
Acute-care beds per million	1.00 (1.00, 1.00)	(0.17, 5.8)	50.0

Model 3: multilevel logistic regression with patient- and cluster-level variables and random intercepts for clusters

*CI* confidence interval, *IOR* interval odds ratio, *POOR* proportion of opposed odds ratios, *ICU* intensive care unit, *HDU* high-dependency care unit, *IMV* invasive mechanical ventilation

### Regional variations in intensive care unit admission

The median number of ICU beds in the analysed regions was 8 (IQR, 0–24), and 49 regions (35.0%) did not have any ICU beds (Supplemental Table 7), which accounted for 8.4% (*n* = 7011/83,346) of the IMV patients in those 49 regions. The ICU admission rates varied widely between the 140 regions, from 0 to 72.1% (median, 28.7%; IQR, 0.9–46.2%). The hospital transfer rate on the day of IMV initiation in regions without ICU beds was 0.6% (*n* = 43/7011). The ICU admission rate increased to ~ 50% in regions with eight ICU beds, after which it plateaued (Fig. 1B). The posterior means of the random effects in Model 3 showed significant differences between regions (Fig. 2B). In Model 1, the general contextual effect of the regional cluster was large, with an ICC of 67.3% and an MOR of 12.0 (Table 2). Accounting for patient-level variables in Model 2 did not change the ICC or MOR, with a PCV of –1.0%. Further adjustment for regional-level variables in Model 3 decreased the ICC and MOR, with a PCV of 86.2%. Among the regional-level variables, regions with ICU beds had a statistically significant and strong association with outcomes (IOR-80%, 9.09–305.48 and POOR, 0.2; Table 3). The full results of Models 2 and 3 for the multilevel analyses with regional clusters are presented in Supplemental Table 8.

### Results of sensitivity analyses for hospitals and regions with at least one ICU bed

After excluding 20,563 patients treated in the 346 hospitals without an ICU bed, 62,783 patients from 200 hospitals were eligible for the sensitivity analysis. Of these, 33,534 (53.4%) were treated in ICU beds on the day of IMV initiation. The posterior means of the random effects in Model 3 showed significant differences between hospitals (Supplemental Fig. 2). In Model 1, the general contextual effect of the hospital cluster was large, with an ICC of 26.0% and MOR of 2.79 (Supplemental Table 9). Accounting for patient-level variables in Model 2 did not change the ICC or MOR, with a PCV of − 4.3%. Further adjustment for hospital-level variables in Model 3 reduced the ICC and MOR, with a PCV of 41.7%. Among the hospital-level variables, no significant associations with outcomes were observed (Supplemental Table 10). The detailed results of Models 2 and 3 for the multilevel analyses of hospital clusters are shown in Supplemental Table 11.

After excluding 7011 patients treated in the 49 regions without an ICU bed, 76,335 patients from 91 regions were eligible for the sensitivity analysis. Of these, 33,599 (44.0%) were treated in ICU beds on the day of IMV initiation. The posterior means of the random effects in Model 3 showed significant differences between regions (Supplemental Fig. 3). In Model 1, the general contextual effect of the regional cluster was large, with an ICC of 27.3% and MOR of 2.89 (Supplemental Table 9). Accounting for patient-level variables in Model 2 did not change the ICC or MOR, with a PCV of 5.7%. Further adjustment for regional-level variables in Model 3 reduced the ICC and MOR, with a PCV of 31.3%. Among the regional-level variables, no significant associations with outcomes were observed (Supplemental Table 10). The detailed results of Models 2 and 3 for the multilevel analyses with regional clusters are presented in Supplemental Table 12.

## Discussion

In this study of > 80,000 patients undergoing IMV in Japan, only 40.4% were treated in ICUs, with significant variations found between hospitals and regions. Among the patients treated outside the ICU on the day of IMV initiation, 97.5% remained outside the ICU during the subsequent IMV. Hospital and regional factors had much greater effects on the decision to treat patients in the ICU than patient-related factors, suggesting that institutional factors and local critical-care healthcare systems play greater roles in terms of patients on IMV being admitted to the ICU. The sensitivity analyses results for hospitals and regions with ICU beds also showed significant variations in ICU admission by hospital and region, with hospital and regional clusters significantly influencing the decision to admit patients in the ICU.

The study used data from Japan, where the number of ICU beds per population is smaller (5.4 per 100,000 population) than in the United States (34.7 beds per 100,000 population), Germany (29.2), and Taiwan (28.5) [23–25]. Consistent with previous reports [3, 5], the ICU admission rate for patients on IMV was found to reach a maximum of 40% in Japan. It is essential to emphasise that our study was based on results from regions with low critical care capacities. A recent study estimated that like Japan, at least 96 countries—particularly those classified as low- and middle-income countries—have ICU bed densities of < 5.0 per 100,000 population. [26–30] Furthermore, there are significant variations in critical care capacities within and across countries worldwide [26–30]. The situation in Japan, which has a small number of ICU beds and a low proportion of ICU admissions, makes it suitable for examining the factors that oppose ICU admissions for patients undergoing IMV.

For hospital clusters, the ICC derived from Model 1 was 82.2%, indicating that 82.2% of the variation in the underlying propensity of patients on IMV to be treated in the ICU was caused by differences between hospitals (without considering the possibility of a different patient-mix composition when estimating inter-hospital variance), while the remaining 17.8% was due to differences between patients. Further adjustment for patient-level variables in Model 2 did not change the ICC and MOR, with a PCV of 2.3%, indicating that patient factors did not explain the variation in the ICU admission rates of patients on IMV. Further adjustment for hospital-level variables in Model 3 decreased the ICC and MOR, with a PCV of 95.2%, indicating that the influence of hospital-level factors significantly contributed to the variability in ICU admission rates for patients on IMV. The same was true for the regional clusters. To the best of our knowledge, this is the first study to quantitatively show that a considerable proportion of the factors that oppose ICU admission for patients receiving IMV can be explained by hospital- or regional-level factors. In other words, the disparities in access to ICU admission for patients undergoing IMV are based largely on hospitals and regions. Given that IMV outside the ICU is associated with a worse prognosis, the results of this study raise several important issues, including health outcome inequalities, healthcare service imbalances, risk of vulnerability to disasters or pandemics, increased burden on healthcare workers, and impacts on the local economy [3, 4].

The disparities we observed in terms of ICU admissions based on hospital and regional factors highlight the need for targeted interventions and policy considerations. Based on our results, the ICU admission rates for patients on IMV in hospitals or regions without ICU beds may represent a potential target for future intervention. As patients on IMV in hospitals and regions without ICU beds accounted for 24.7% (*n* = 20,563/83,346) and 8.4% (*n* = 7011/83,346) of all patients receiving IMV, respectively, many patients would benefit from these interventions. Possible policy interventions include establishing grants for hospitals and regions to add new ICU beds, increasing reimbursements for IMV management in ICUs, and developing new reimbursements for interhospital transfers of patients so that they can be admitted to ICUs. Another perspective is that both the hospital- and regional-level number of ICU beds may be a concern based on the odds ratio found in this study; however, it was not found to be statistically significant in terms of IOR-80% and POOR. This study also indicates that factors other than the hospital- and regional-level variables we observed may exist. One possibility is variations in management practices for ICU beds, which suggests that ICU management may benefit from greater standardisation, via national-level guidelines or changing the reimbursement criteria for ICU admissions. Future research is warranted to address unmeasured hospital- and regional-level factors that influence variations in ICU admissions with the goal of optimising critical care resources and improving patient outcomes.

This study was subject to a few key limitations worth noting. First, it focused on Japan; therefore, the generalisability of our findings to other countries with different healthcare systems and resource allocations may be limited. Second, 199 of the 339 secondary medical areas in Japan were excluded. However, the impact of these excluded cohorts was negligible, as is shown in Supplementary Table 3, which suggests that the results do have internal validity. Third, the detailed reasons for a lack of admission to the ICU were not investigated at the individual patient level. Furthermore, it was unknown where the patient was intubated prior to admission. Fourth, factors that were not measured at the patient, hospital, and regional levels may have influenced our results. Fifth, this study did not examine ICU admissions during or after the coronavirus disease 2019 pandemic, which is a topic for future research.

## Conclusions

This study quantitatively identified variations in the ICU admission rates of patients in Japan undergoing IMV, at both the hospital and regional levels. These findings have important implications for healthcare policymakers in terms of future targeted interventions for ICU resource allocation. Future research should examine the institutional and regional factors that influence these variations, with the ultimate goal of optimising critical care delivery and improving patient outcomes.

### Supplementary Information

Supplementary Material 1.

## Data Availability

The data used in the manuscript will not be made available because the datasets analysed are not publicly available, owing to contracts with the hospitals that provided the data.

## Abbreviations

IMV: Invasive mechanical ventilation
ICU: Intensive care unit
DPC: Diagnosis procedure combination inpatient
ICD-10: International Classification of Diseases, Tenth Revision
HDU: High-dependency care unit
ICC: Intraclass correlation coefficient
MOR: Median odds ratio
PCV: Proportional change in variance
AUC: Area under the receiver operating characteristic curve
IOR: Interval odds ratio
POOR: Proportion of opposed odds ratio
IQR: Interquartile range
SD: Standard deviation
