# Supplementary material for: Hospital and regional variations in intensive care unit admission for patients with invasive mechanical ventilation
Source: J Intensive Care. 2024 Jun 5;12:21. doi: 10.1186/s40560-024-00736-0 (PMC11155017; doi:10.1186/s40560-024-00736-0)
Supplement: Supplementary file 1 — Supplementary Material 1. [file 40560_2024_736_MOESM1_ESM.docx]

***Supplemental Materials***

**Hospital and regional variations in intensive care unit admission for patients with invasive mechanical ventilation**

**Supplemental Figure 1. Patient recruitment flow chart.**

**
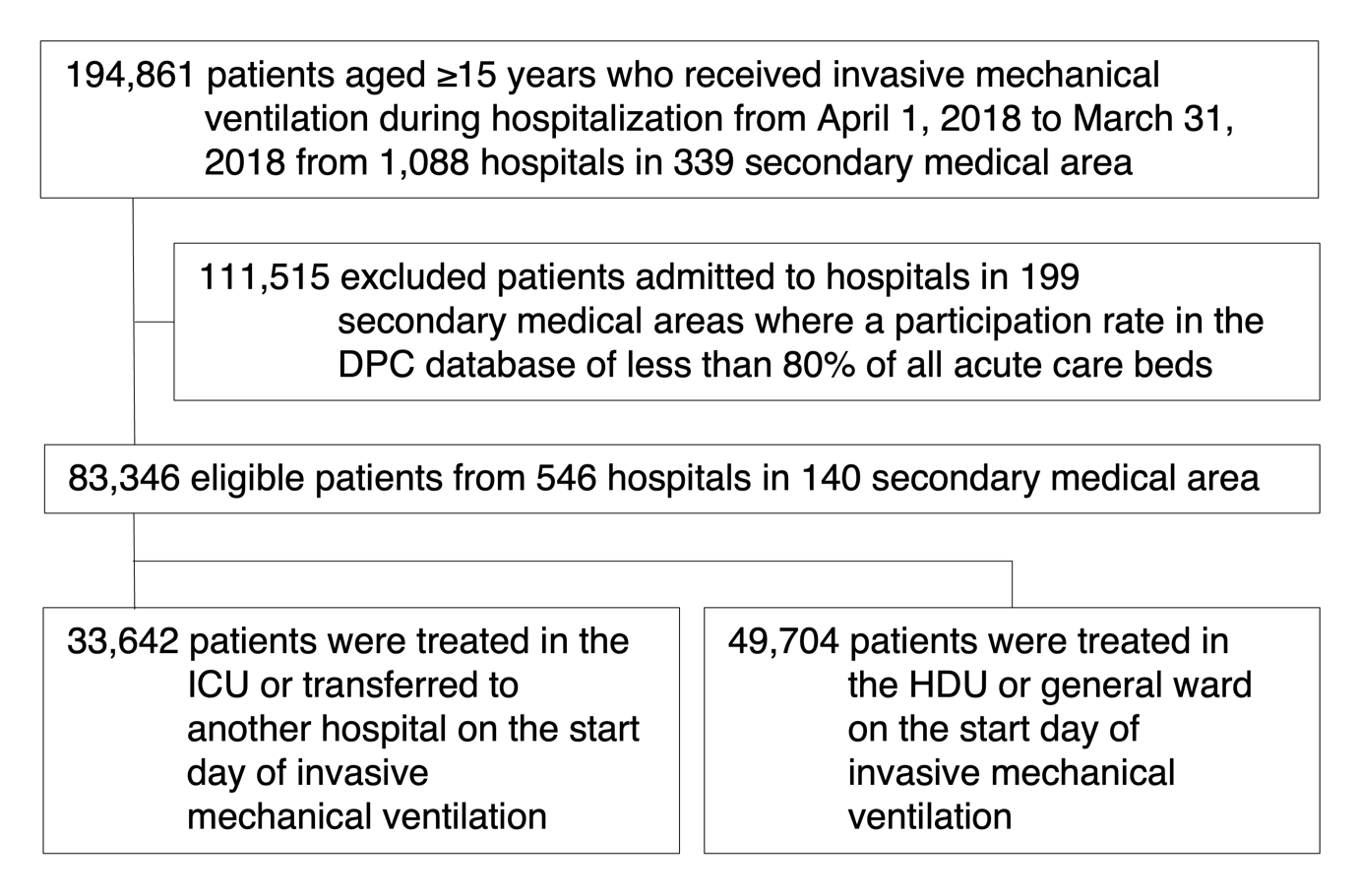
**

DPC, Diagnosis Procedure Combination; ICU, intensive care unit; HDU, high-dependency care unit.

**Supplemental Figure 2. Posterior means of random effects to evaluate the cluster variations in hospital using Model 3 in the sensitivity analysis of hospitals with at least one ICU beds.**


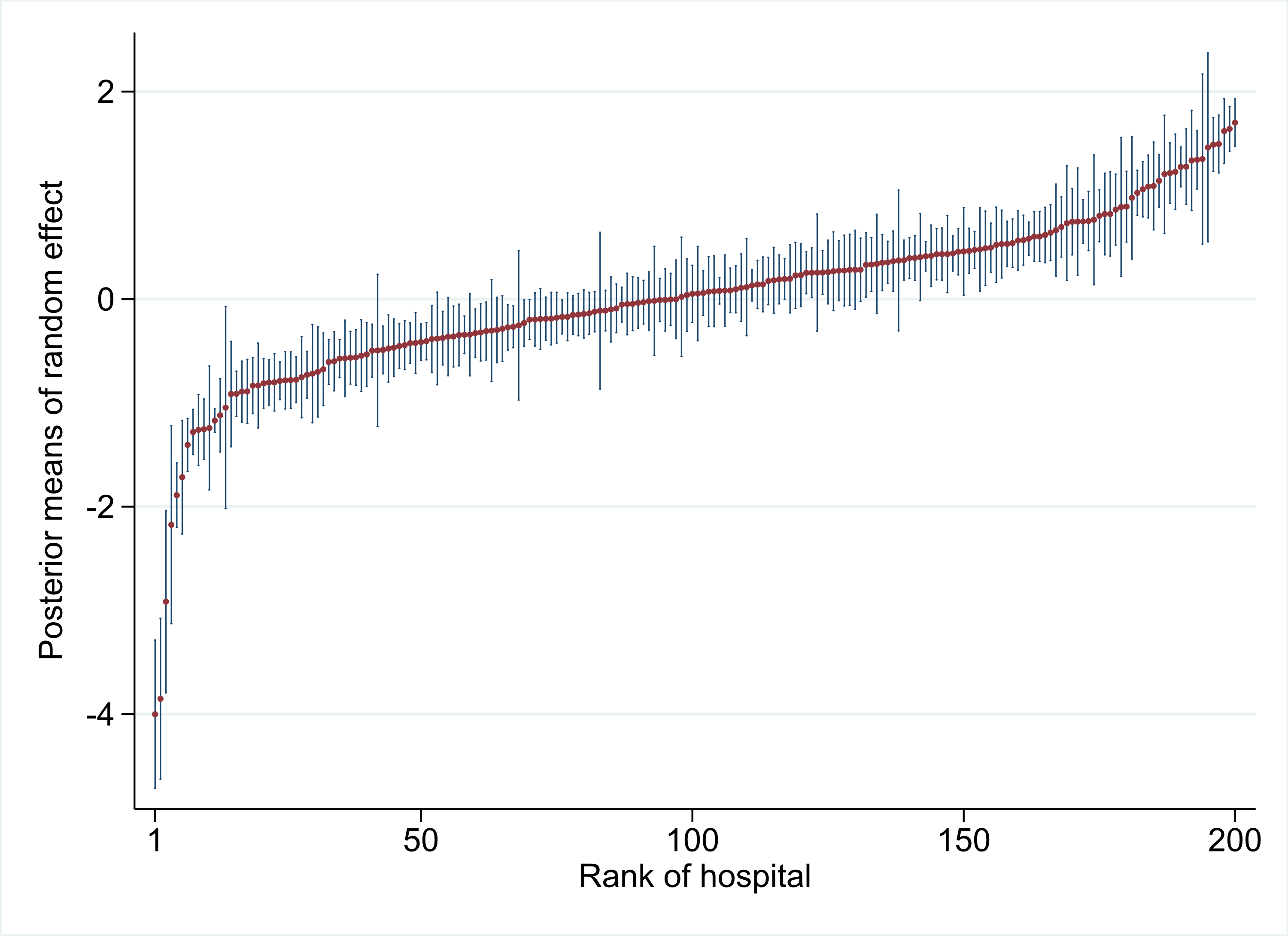


Model 3 was a multilevel logistic regression model with patient-level covariates, cluster-level variables, and random intercepts for the clusters.

**Supplemental Figure 3. Posterior means of random effects to evaluate the cluster variations in secondary medical areas using Model 3 in the sensitivity analysis of secondary medical areas with at least one ICU beds.**


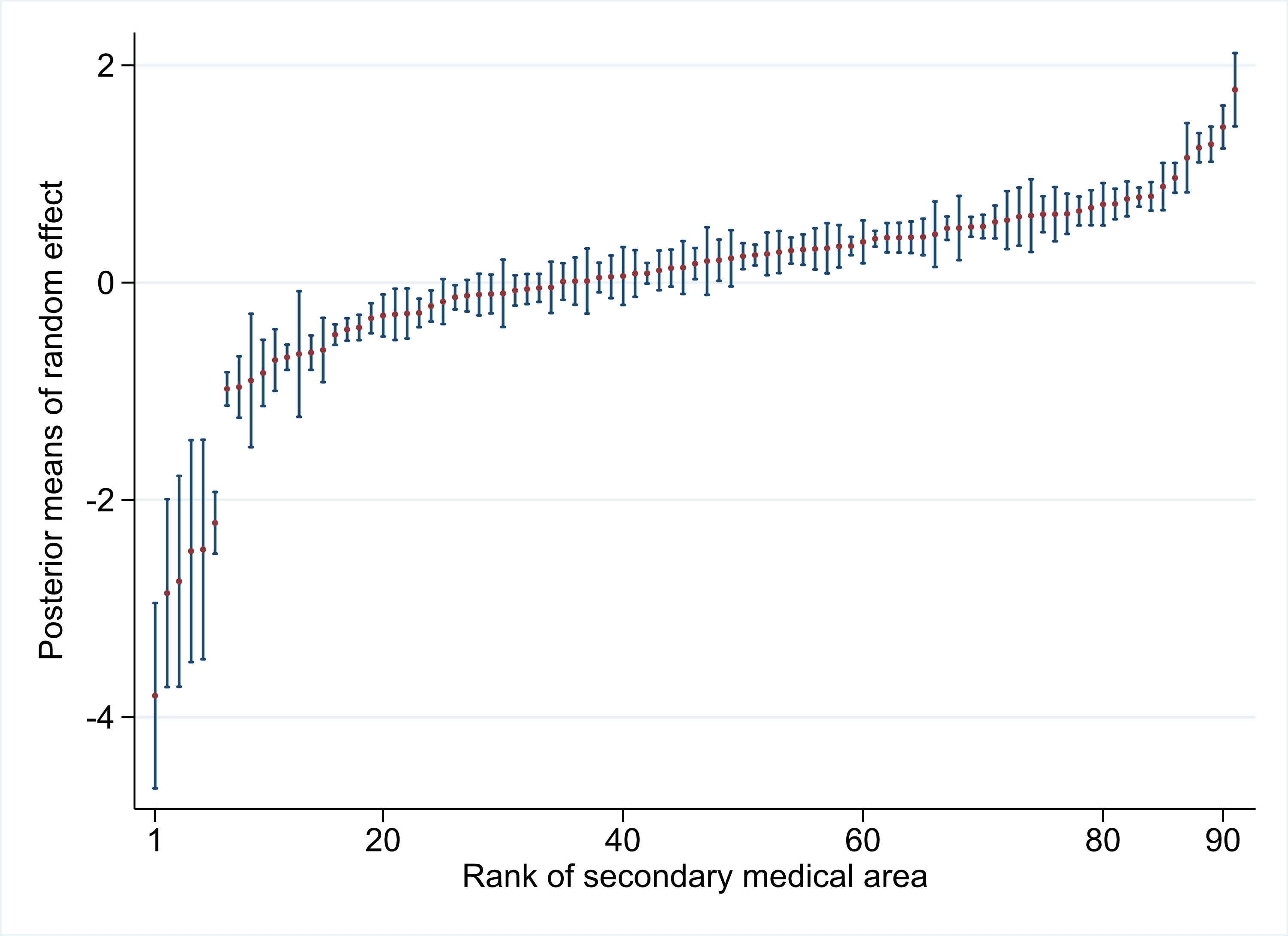


Model 3 was a multilevel logistic regression model with patient-level covariates, cluster-level variables, and random intercepts for the clusters.

**Supplemental Table 1. Japanese medical procedure codes used to define acute care beds in the ICU, HDU, and general wards.**

|  | **Japanese** |  | **Cost** |  |  |
| --- | --- | --- | --- | --- | --- |
|  | **procedure** |  | **per day,** | **Intensivist** | **Nurse-to-patient** |
| **Name** | **Code** | **Description** | **yen*** | **staffing** | **ratio** |
| ICU | A3011 | ICU management fee 1 | 142,110 | ≥ 2 | 1 to 2 |
| ICU | A3012 | ICU management fee 2 | 142,110 | ≥ 2 | 1 to 2 |
| ICU | A3013 | ICU management fee 3 | 96,970 | – | 1 to 2 |
| ICU | A3014 | ICU management fee 4 | 96,970 | – | 1 to 2 |
| ICU | A3002 | Emergency and critical care unit management fee 2 | 106,860 | – | 1 to 2 |
| ICU | A3004 | Emergency and critical care unit management fee 4 | 106,860 | – | 1 to 2 |
| HDU | A3001 | Emergency and critical care unit management fee 1 | 92,500 | – | 1 to 4 |
| HDU | A3003 | Emergency and critical care unit management fee 3 | 92,500 | – | 1 to 4 |
| HDU | A301-21 | HDU management fee 1 | 68,550 | – | 1 to 4 |
| HDU | A301-22 | HDU management fee 2 | 42,240 | – | 1 to 5 |
| General ward | A1001 | Acute general inpatient fee 1 | 16,500 | – | 1 to 7 |
| General ward | A1001 | Acute general inpatient fee 2 | 16,190 | – | 1 to 10 |
| General ward | A1001 | Acute general inpatient fee 3 | 15,450 | – | 1 to 10 |
| General ward | A1001 | Acute general inpatient fee 4 | 14,400 | – | 1 to 10 |
| General ward | A1001 | Acute general inpatient fee 5 | 14,290 | – | 1 to 10 |
| General ward | A1001 | Acute general inpatient fee 6 | 13,820 | – | 1 to 10 |
| General ward | A1041 | General inpatient fee with specific function 1 | 17,180 | – | 1 to 7 |
| General ward | A1041 | General inpatient fee with specific function 2 | 14,380 | – | 1 to 10 |

*Cost per day for the first seven days after admission.

ICU, intensive care unit; HDU, high-dependency care unit.

**Supplemental Table 2. Details of our multilevel logistic regression model.**

| **Model** | **Dependent variables** | **Random effect** |
| --- | --- | --- |
| **Hospital-level** | |  |
| Model 1 | None | Random intercepts for hospital |
| Model 2 | Patient-level variables | Random intercepts for hospital |
| Model 3 | Patient-level variables + hospital-level variables | Random intercepts for hospital |
| **Regional-level** | |  |
| Model 1 | None | Random intercepts for region |
| Model 2 | Patient-level variables | Random intercepts for region |
| Model 3 | Patient-level variables + regional-level variables | Random intercepts for region |

Patient-level variables included age, sex, body mass index at admission, Charlson Comorbidity Index score, cognitive function before admission, long-term care needs before admission, home medical care before admission, location before hospitalisation, admission on a weekend, ambulance use, emergency admission, surgery under general anaesthesia before invasive mechanical ventilation, cardiopulmonary resuscitation on the start day of invasive mechanical ventilation, length of hospital stay before invasive mechanical ventilation, primary diagnoses at admission, and geodetic distance from home to the nearest ICU. Hospital-level variables included hospitals with ICU beds, number of ICU beds, number of HDU beds, number of acute care beds, academic hospitals, tertiary emergency hospitals, annual number of ambulances, and annual hospital case volume of invasive mechanical ventilation. Regional-level variables were regions with ICU beds, number of ICU beds, HDU beds, and acute-care beds per 100,000 people.

ICU, intensive care unit; HDU, high-dependency care unit.

**Supplemental Table 3. Patient-, hospital-, and regional-level characteristics of the patients included and excluded from this study.**

|  | **Patients** | **Patients** |  |
| --- | --- | --- | --- |
|  | **included** | **excluded** |  |
| **Variables** | **n = 83,346** | **n = 111,515** | **SMD** |
| ICU admission on the day of IMV, n (%) | 33,498 (40.2) | 53,423 (47.9) | – |
| **Patient-level** |  |  |  |
| Age, years, mean (SD) | 72.1 (15.4) | 71.2 (15.8) | –6 |
| Male, n (%) | 50,470 (60.6) | 67,783 (60.8) | 0 |
| BMI at admission, kg/m^2^, n (%) |  |  |  |
| < 18.5 | 13,844 (16.6) | 17,972 (16.1) | –1 |
| 18.5–24.9 | 39,722 (47.7) | 50,987 (45.7) | –4 |
| 25.0–29.9 | 12,520 (15.0) | 16,089 (14.4) | –2 |
| ≥ 30.0 | 3,688 (4.4) | 5,044 (4.5) | 0 |
| Missing | 13,572 (16.3) | 21,423 (19.2) | 8 |
| CCI, mean (SD) | 1.2 (1.6) | 1.2 (1.5) | –4 |
| Cognitive function, n (%) |  |  |  |
| No dementia | 65,421 (78.5) | 90,677 (81.3) | 7 |
| Mild dementia | 9,628 (11.6) | 11,363 (10.2) | –4 |
| Moderate/severe dementia | 8,297 (10.0) | 9,475 (8.5) | –5 |
| Long-term care-needs, n (%) |  |  |  |
| No care-needs | 83,043 (99.6) | 111,164 (99.7) | 1 |
| SL1-2 & CNL1-2 | 125 (0.1) | 118 (0.1) | –1 |
| CNL3-5 | 178 (0.2) | 233 (0.2) | 0 |
| Home medical care, n (%) | 5,524 (6.6) | 7,509 (6.7) | 0 |
| Admission on a weekend, n (%) | 19,886 (23.9) | 26,544 (23.8) | 0 |
| Location before admission, n (%) |  |  |  |
| Home | 71,142 (85.4) | 96,142 (86.2) | 3 |
| Other hospitals | 7,015 (8.4) | 9,003 (8.1) | –2 |
| Nursing home | 5,189 (6.2) | 6,370 (5.7) | –2 |
| Ambulance use, n (%) | 50,843 (61.0) | 69,975 (62.7) | 4 |
| Emergency admission, n (%) | 66,927 (80.3) | 88,684 (79.5) | –2 |
| Surgery, n (%) | 9,863 (11.8) | 14,443 (13.0) | 4 |
| CPR, n (%) | 16,510 (19.8) | 22,711 (20.4) | 1 |
| Length of stay before IMV, n (%) |  |  |  |
| On the day of admission | 40,903 (49.1) | 55,182 (49.5) | 1 |
| On the next day of admission | 12,345 (14.8) | 16,353 (14.7) | 0 |
| Day 3–6 | 14,358 (17.2) | 19,951 (17.9) | 2 |
| Day 7– | 15,740 (18.9) | 20,029 (18.0) | –3 |
| Primary diagnoses, n (%) |  |  |  |
| Acute heart failure | 12,713 (15.3) | 15,613 (14.0) | –4 |
| Post cardiac arrest | 10,206 (12.2) | 18,222 (16.3) | 12 |
| Acute coronary syndrome | 7,315 (8.8) | 9,882 (8.9) | 1 |
| Stroke | 7,518 (9.0) | 9,589 (8.6) | –1 |
| Cancer | 6,942 (8.3) | 8,687 (7.8) | –2 |
| Aortic dissection or aneurysm | 5,747 (6.9) | 7,838 (7.0) | 1 |
| Abdominal diseases | 5,725 (6.9) | 7,264 (6.5) | –2 |
| Pneumonia | 5,290 (6.3) | 6,746 (6.0) | –1 |
| Trauma | 4,474 (5.4) | 5,490 (4.9) | –2 |
| Sepsis | 3,179 (3.8) | 4,496 (4.0) | 1 |
| Aspiration | 3,303 (4.0) | 3,871 (3.5) | –3 |
| Chronic lower respiratory diseases | 1,859 (2.2) | 2,381 (2.1) | –1 |
| Home to nearest ICU, km, median (IQR) | 5.1 (2.4–11.9) | 3.1 (1.7–6.6) | –22 |
| **Hospital-level** |  |  |  |
| Hospitals with ICU beds, n (%) | 62,783 (75.3) | 89,160 (80.0) | 10 |
| ICU beds, median (IQR) | 8 (3–16) | 10 (0–18) | 9 |
| HDU beds, median (IQR) | 15 (4–25) | 10 (0–24) | –15 |
| Acute-care beds, median (IQR) | 490 (300–650) | 477 (291–676) | –9 |
| Academic hospital, n (%) | 16,360 (19.6) | 22,079 (19.8) | 1 |
| Tertiary emergency hospital, n (%) | 46,052 (55.3) | 52,615 (47.2) | –15 |
| Ambulance cases, median (IQR) | 3,921 (2,200–5,739) | 4,615 (2,774–6,511) | 24 |
| IMV cases, median (IQR) | 335 (173–487) | 398 (200–597) | 18 |
| **Regional-level** |  |  |  |
| Regions with ICU beds, n (%) | 76,335 (91.6) | 105,047 (94.2) | 10 |
| ICU beds per million, median (IQR) | 6.1 (3.9–8.6) | 6.5 (4.5–8.9) | 16 |
| HDU beds per million, median (IQR) | 11.8 (8.2–15.3) | 9.9 (7.7–13.7) | –18 |
| Acute-care beds per million, median (IQR) | 621 (501–692) | 542 (454–704) | –9 |

ICU, intensive care unit; HDU, high-dependency care unit; SMD, standardised mean difference; SD, standard deviation; BMI, body mass index; CCI, Charlson Comorbidity Index; SL, support level; CNL, care needs level; CPR, cardiopulmonary resuscitation; IMV, invasive mechanical ventilation; IQR, interquartile range.

**Supplemental Table 4. Clinical outcomes of patients who underwent invasive mechanical ventilation in the ICU, HDU, or general ward.**

|  | **ICU** | **HDU/ward** |
| --- | --- | --- |
| **Variables** | **n = 33,642** | **n = 49,704** |
| ICU admission after the day of IMV, n (%) | – | 1,231 (2.5) |
| HDU admission after the day of IMV, n (%) | 6,210 (18.5) | 13,623 (27.4) |
| In-hospital mortality, n (%) | 8,297 (24.7) | 24,346 (49.0) |
| Length of hospital stay, median (IQR) | 22.0 (12.0–40.0) | 11.0 (1.0–28.0) |
| Length of IMV, median (IQR) | 2.0 (1.0–7.0) | 2.0 (1.0–7.0) |
| Length of ICU stay, median (IQR) | 4.0 (2.0–8.0) | – |
| Total hospitalization costs, million yen, median (IQR) | 3.5 (1.7–5.6) | 1.0 (0.2–2.3) |

ICU, intensive care unit; HDU, high-dependency care unit; IMV, invasive mechanical ventilation; IQR, interquartile range.

**Supplemental Table 5. Characteristics of 546 hospitals included in the study and a comparison of hospitals with and without ICU beds.**

|  |  | **Hospitals with** | **Hospitals without** |
| --- | --- | --- | --- |
|  | **Overall** | **ICU beds** | **ICU beds** |
| **Variables** | **(n = 546)** | **(n = 200)** | **(n = 346)** |
| Hospitals with ICU beds, n (%) | 200 (36.6) | 200 (100.0) | 0 (0.0) |
| ICU beds, median (IQR) | 0 (0–6) | 8 (6–13) | 0 (0–0) |
| HDU beds, median (IQR) | 0 (0–12) | 12 (0–24) | 0 (0–4) |
| Acute-care beds, median (IQR) | 226 (118–408) | 482 (357–627) | 143 (86–226) |
| Academic hospital, n (%) | 36 (6.6) | 36 (18.0) | 0 (0.0) |
| Tertiary emergency hospital, n (%) | 113 (20.7) | 101 (50.5) | 12 (3.5) |
| Ambulance cases, median (IQR) | 1,834 (791–3,404) | 3,701 (2,515–5,255) | 1,083 (505–1,997) |
| IMV cases, median (IQR) | 78 (27–218) | 285 (180–422) | 40 (16–80) |

ICU, intensive care unit; HDU, high-dependency care unit; IQR, interquartile range; IMV, invasive mechanical ventilation.

**Supplemental Table 6. Results of multilevel logistic regression analyses of Models 2 and 3 with hospital clusters.**

|  | **Model 2** |  | **Model 3** |  |
| --- | --- | --- | --- | --- |
|  | **Odds ratio** |  | **Odds ratio** |  |
| **Variables** | **(95% CI)** | **P** | **(95% CI)** | **P** |
| **Patient-level** |  |  |  |  |
| Age | 0.99 (0.99, 0.99) | < 0.001 | 0.99 (0.99, 1.00) | < 0.001 |
| Male | 0.96 (0.92, 1.00) | 0.037 | 0.96 (0.92, 1.00) | 0.042 |
| BMI at admission |  |  |  |  |
| < 18.5 | 0.76 (0.72, 0.80) | < 0.001 | 0.76 (0.72, 0.81) | < 0.001 |
| 18.5–24.9 | Ref. |  | Ref. |  |
| 25.0–29.9 | 1.03 (0.98, 1.09) | 0.282 | 1.03 (0.98, 1.09) | 0.271 |
| ≥ 30.0 | 0.84 (0.77, 0.92) | < 0.001 | 0.84 (0.77, 0.92) | < 0.001 |
| Missing | 0.49 (0.46, 0.53) | < 0.001 | 0.50 (0.46, 0.53) | < 0.001 |
| CCI | 1.03 (1.01, 1.04) | < 0.001 | 1.03 (1.01, 1.04) | < 0.001 |
| Cognitive function |  |  |  |  |
| No dementia | Ref. |  | Ref. |  |
| Mild dementia | 0.88 (0.83, 0.94) | < 0.001 | 0.89 (0.83, 0.95) | < 0.001 |
| Moderate/severe dementia | 0.91 (0.84, 0.98) | 0.017 | 0.92 (0.85, 0.99) | 0.031 |
| Long-term care-needs |  |  |  |  |
| No care-needs | Ref. |  | Ref. |  |
| SL1-2 & CNL1-2 | 0.91 (0.42, 1.97) | 0.813 | 0.94 (0.43, 2.06) | 0.884 |
| CNL3-5 | 1.30 (0.63, 2.68) | 0.470 | 1.57 (0.73, 3.36) | 0.246 |
| Home medical care | 0.48 (0.44, 0.52) | < 0.001 | 0.48 (0.44, 0.52) | < 0.001 |
| Weekend admission | 1.14 (1.09, 1.19) | < 0.001 | 1.14 (1.09, 1.19) | < 0.001 |
| Location before admission |  |  |  |  |
| Home | Ref. |  | Ref. |  |
| Other hospitals | 1.12 (1.05, 1.20) | 0.001 | 1.12 (1.05, 1.21) | 0.001 |
| Nursing home | 0.73 (0.67, 0.80) | < 0.001 | 0.73 (0.67, 0.81) | < 0.001 |
| Ambulance use | 1.35 (1.28, 1.43) | < 0.001 | 1.34 (1.27, 1.42) | < 0.001 |
| Emergency admission | 0.64 (0.60, 0.68) | < 0.001 | 0.64 (0.60, 0.69) | < 0.001 |
| Surgery | 2.71 (2.54, 2.89) | < 0.001 | 2.69 (2.52, 2.87) | < 0.001 |
| CPR | 0.43 (0.40, 0.46) | < 0.001 | 0.43 (0.41, 0.46) | < 0.001 |
| Length of stay before IMV |  |  |  |  |
| On the day of admission | Ref. |  | Ref. |  |
| On the next day of admission | 1.73 (1.63, 1.84) | < 0.001 | 1.72 (1.62, 1.83) | < 0.001 |
| Day 3–6 | 1.71 (1.60, 1.82) | < 0.001 | 1.70 (1.60, 1.81) | < 0.001 |
| Day 7– | 1.40 (1.31, 1.49) | < 0.001 | 1.40 (1.31, 1.49) | < 0.001 |
| Primary diagnoses |  |  |  |  |
| Acute heart failure | 0.69 (0.65, 0.74) | < 0.001 | 0.70 (0.65, 0.74) | < 0.001 |
| Post cardiac arrest | 1.13 (1.05, 1.23) | 0.002 | 1.13 (1.05, 1.22) | 0.002 |
| Acute coronary syndrome | 0.78 (0.73, 0.84) | < 0.001 | 0.79 (0.73, 0.85) | < 0.001 |
| Stroke | 2.99 (2.78, 3.22) | < 0.001 | 2.97 (2.76, 3.19) | < 0.001 |
| Cancer | 1.14 (1.05, 1.23) | < 0.001 | 1.14 (1.06, 1.23) | 0.001 |
| Aortic dissection or aneurysm | 3.18 (2.92, 3.46) | < 0.001 | 3.15 (2.90, 3.43) | < 0.001 |
| Abdominal diseases | 1.71 (1.57, 1.85) | < 0.001 | 1.71 (1.57, 1.85) | < 0.001 |
| Pneumonia | 0.56 (0.51, 0.61) | < 0.001 | 0.56 (0.51, 0.62) | < 0.001 |
| Trauma | 1.15 (1.06, 1.26) | 0.001 | 1.16 (1.06, 1.26) | 0.001 |
| Sepsis | 2.14 (1.94, 2.37) | < 0.001 | 2.14 (1.94, 2.37) | < 0.001 |
| Aspiration | 0.71 (0.63, 0.79) | < 0.001 | 0.71 (0.64, 0.79) | < 0.001 |
| Chronic respiratory diseases | 0.39 (0.33, 0.45) | < 0.001 | 0.39 (0.34, 0.46) | < 0.001 |
| Home to nearest ICU, km | 1.00 (1.00, 1.01) | < 0.001 | 1.01 (1.00, 1.01) | < 0.001 |
| **Hospital-level** |  |  |  |  |
| Hospitals with ICU beds |  |  | 239 (172, 332) | < 0.001 |
| ICU beds |  |  | 1.10 (1.08, 1.12) | < 0.001 |
| HDU beds |  |  | 0.99 (0.98, 1.00) | 0.17 |
| Acute-care beds |  |  | 1.00 (1.00, 1.00) | 0.004 |
| Academic hospital |  |  | 1.13 (0.70, 1.80) | 0.619 |
| Tertiary emergency hospital |  |  | 0.62 (0.45, 0.84) | 0.002 |
| Ambulance cases |  |  | 1.00 (1.00, 1.00) | 0.466 |
| IMV cases |  |  | 1.00 (1.00, 1.00) | 0.041 |

Model 2: multilevel logistic regression with patient-level covariates and random intercepts for clusters. Model 3: multilevel logistic regression with patient-level variables, cluster-level variables, and random intercepts for clusters.

ICU, intensive care unit; HDU, high-dependency care unit; SMD, standardised mean difference; SD, standard deviation; BMI, body mass index; CCI, Charlson Comorbidity Index; SL, support level; CNL, care needs level; CPR, cardiopulmonary resuscitation; IMV, invasive mechanical ventilation; IQR, interquartile range.

**Supplemental Table 7. Characteristics of 140 secondary medical areas included in the study.**

|  | **Overall** | **Regions with ICU beds** | **Regions without ICU beds** |
| --- | --- | --- | --- |
| **Variables** | **(n = 140)** | **(n = 91)** | **(n = 49)** |
| Covering population, median (IQR) | 206,105 (106,828–416,785) | 315,174 (191,962–527,700) | 82,776 (57,075–137,618) |
| Hospitals with ICU beds, n (%) | 91 (65.0) | 91 (100.0) | 0 (0.0) |
| ICU beds in region, median (IQR) | 8 (0–24) | 18 (8–35) | 0 (0–0) |
| HDU beds in region, median (IQR) | 17 (2–48) | 36 (15–65) | 0 (0–10) |
| Acute-care beds in region, median (IQR) | 1,133 (569–2425) | 1,845 (1,040–3,299) | 462 (296–680) |
| ICU beds per million, median (IQR) | 3.7 (0.0–7.0) | 5.7 (3.9–8.6) | 0.0 (0.0–0.0) |
| HDU beds per million, median (IQR) | 8.9 (0.9–13.3) | 11.0 (6.2–14.4) | 0.0 (0.0–11.6) |
| Acute-care beds per million, median (IQR) | 573 (463–680) | 603 (475–693) | 558 (434–654) |

IQR, interquartile range; ICU, intensive care unit; HDU, high-dependency care unit.

**Supplemental Table 8. Results of multilevel logistic regression analyses of Models 2 and 3 with regional clusters.**

|  | **Model 2** |  | **Model 3** |  |
| --- | --- | --- | --- | --- |
|  | **Odds ratio** |  | **Odds ratio** |  |
| **Variables** | **(95% CI)** | **P** | **(95% CI)** | **P** |
| **Patient-level** |  |  |  |  |
| Age | 0.99 (0.99, 0.99) | < 0.001 | 0.99 (0.99, 0.99) | < 0.001 |
| Male | 0.96 (0.93, 0.99) | 0.023 | 0.96 (0.93, 0.99) | 0.024 |
| BMI at admission |  |  |  |  |
| < 18.5 | 0.74 (0.71, 0.78) | < 0.001 | 0.74 (0.71, 0.78) | < 0.001 |
| 18.5–24.9 | Ref. |  | Ref. |  |
| 25.0–29.9 | 1.04 (0.99, 1.09) | 0.118 | 1.04 (0.99, 1.09) | 0.119 |
| ≥30.0 | 0.88 (0.81, 0.95) | 0.002 | 0.88 (0.81, 0.95) | 0.002 |
| Missing | 0.50 (0.47, 0.53) | < 0.001 | 0.50 (0.47, 0.53) | < 0.001 |
| CCI | 1.03 (1.01, 1.04) | < 0.001 | 1.03 (1.01, 1.04) | < 0.001 |
| Cognitive function |  |  |  |  |
| No dementia | Ref. |  | Ref. |  |
| Mild dementia | 0.87 (0.82, 0.92) | < 0.001 | 0.87 (0.82, 0.92) | < 0.001 |
| Moderate/severe dementia | 0.79 (0.74, 0.84) | < 0.001 | 0.79 (0.74, 0.84) | < 0.001 |
| Long-term care-needs |  |  |  |  |
| No care-needs | Ref. |  | Ref. |  |
| SL1-2 & CNL1-2 | 0.37 (0.22, 0.63) | < 0.001 | 0.37 (0.22, 0.63) | < 0.001 |
| CNL3-5 | 0.27 (0.16, 0.46) | < 0.001 | 0.27 (0.16, 0.46) | < 0.001 |
| Home medical care | 0.46 (0.42, 0.49) | < 0.001 | 0.46 (0.42, 0.49) | < 0.001 |
| Weekend admission | 1.14 (1.09, 1.18) | < 0.001 | 1.14 (1.09, 1.18) | < 0.001 |
| Location before admission |  |  |  |  |
| Home | Ref. |  | Ref. |  |
| Other hospitals | 1.12 (1.05, 1.18) | < 0.001 | 1.12 (1.05, 1.18) | < 0.001 |
| Nursing home | 0.71 (0.66, 0.78) | < 0.001 | 0.71 (0.66, 0.78) | < 0.001 |
| Ambulance use | 1.57 (1.49, 1.64) | < 0.001 | 1.57 (1.49, 1.64) | < 0.001 |
| Emergency admission | 0.62 (0.58, 0.66) | < 0.001 | 0.62 (0.59, 0.66) | < 0.001 |
| Surgery | 2.69 (2.55, 2.85) | < 0.001 | 2.69 (2.55, 2.84) | < 0.001 |
| CPR | 0.39 (0.37, 0.42) | < 0.001 | 0.39 (0.37, 0.42) | < 0.001 |
| Length of stay before IMV |  |  |  |  |
| On the day of admission | Ref. |  | Ref. |  |
| On the next day of admission | 1.62 (1.54, 1.71) | < 0.001 | 1.62 (1.54, 1.71) | < 0.001 |
| Day 3–6 | 1.70 (1.61, 1.79) | < 0.001 | 1.69 (1x61, 1.79) | < 0.001 |
| Day 7– | 1.35 (1.28, 1.42) | < 0.001 | 1.35 (1.28, 1.42) | < 0.001 |
| Primary diagnoses |  |  |  |  |
| Acute heart failure | 0.73 (0.69, 0.77) | < 0.001 | 0.73 (0.69, 0.77) | < 0.001 |
| Post cardiac arrest | 1.45 (1.35, 1.55) | < 0.001 | 1.45 (1.35, 1.55) | < 0.001 |
| Acute coronary syndrome | 0.80 (0.75, 0.86) | < 0.001 | 0.80 (0.75, 0.86) | < 0.001 |
| Stroke | 2.69 (2.52, 2.86) | < 0.001 | 2.68 (2.52, 2.85) | < 0.001 |
| Cancer | 1.11 (1.04, 1.18) | 0.002 | 1.11 (1.04, 1.18) | 0.001 |
| Aortic dissection or aneurysm | 3.33 (3.09, 3.58) | < 0.001 | 3.32 (3.09, 3.57) | < 0.001 |
| Abdominal diseases | 1.39 (1.30, 1.49) | < 0.001 | 1.39 (1.30, 1.49) | < 0.001 |
| Pneumonia | 0.52 (0.48, 0.56) | < 0.001 | 0.52 (0.48, 0.56) | < 0.001 |
| Trauma | 1.20 (1.11, 1.30) | < 0.001 | 1.20 (1.11, 1.30) | < 0.001 |
| Sepsis | 2.09 (1.92, 2.28) | < 0.001 | 2.09 (1.92, 2.28) | < 0.001 |
| Aspiration | 0.72 (0.66, 0.80) | < 0.001 | 0.72 (0.66, 0.80) | < 0.001 |
| Chronic respiratory diseases | 0.36 (0.31, 0.41) | < 0.001 | 0.36 (0.31, 0.41) | < 0.001 |
| Home to nearest ICU, km | 1.00 (1.00, 1.00) | 0.008 | 1.00 (1.00, 1.00) | 0.003 |
| **Regional-level** |  |  |  |  |
| Regions with ICU beds |  |  | 52.7 (29.1, 95.3) | < 0.001 |
| ICU beds per million |  |  | 1.11 (1.06, 1.17) | < 0.001 |
| HDU beds per million |  |  | 1.00 (0.98, 1.03) | 0.983 |
| Acute-care beds per million |  |  | 1.00 (1.00, 1.00) | 0.741 |

Model 2: multilevel logistic regression with patient-level covariates and random intercepts for clusters. Model 3: multilevel logistic regression with patient-level variables, cluster-level variables, and random intercepts for clusters.

ICU, intensive care unit; HDU, high-dependency care unit; SMD, standardised mean difference; SD, standard deviation; BMI, body mass index; CCI, Charlson Comorbidity Index; SL, support level; CNL, care needs level; CPR, cardiopulmonary resuscitation; IMV, invasive mechanical ventilation; IQR, interquartile range.

**Supplemental Table 9**. **General contextual effects of hospital- and regional-level variables in the sensitivity analyses in hospitals and regions with at least one ICU beds.**

| **Statistic** | **Model 1** | **Model 2** | **Model 3** |
| --- | --- | --- | --- |
| **Hospital-level** |  |  |  |
| ICC (%) | 26.0 (22.0, 30.4) | 26.8 (22.8, 31.3) | 17.6 (14.5, 21.1) |
| MOR | 2.79 (2.48, 3.10) | 2.85 (2.53, 3.17) | 2.22 (2.02, 2.42) |
| PCV (%) |  |  |  |
| Model 1 and 2 | Ref. | -4.3 | – |
| Model 2 and 3 | – | Ref. | 41.7 |
| AUC | 0.725 | 0.817 | 0.817 |
| Difference in AUCs |  |  |  |
| Model 1 and 2 | Ref. | 0.092 | – |
| Model 2 and 3 | – | Ref. | 0 |
| **Regional-level** |  |  |  |
| ICC (%) | 27.3 (21.0, 34.8) | 26.2 (20.1, 33.4) | 20.5 (15.3, 26.9) |
| MOR | 2.89 (2.36, 3.42) | 2.80 (2.30, 3.30) | 2.41 (2.03, 2.79) |
| PCV (%) |  |  |  |
| Model 1 and 2 | Ref. | 5.7 | – |
| Model 2 and 3 | – | Ref. | 31.3 |
| AUC | 0.666 | 0.793 | 0.793 |
| Difference in AUCs |  |  |  |
| Model 1 and 2 | Ref. | 0.127 | – |
| Model 2 and 3 | – | Ref. | 0 |

Model 1: multilevel logistic regression with random intercepts for clusters; Model 2: multilevel logistic regression with patient-level covariates and random intercepts for clusters; and Model 3: multilevel logistic regression with patient-level variables, cluster-level variables, and random intercepts for clusters.

ICU, intraclass correlation coefficient; ICC, intensive care unit; MOR, median odds ratio; PCV, proportional change in variance; AUC, area under the receiver operating characteristic curve.

**Supplemental Table 10. Specific contextual effects of hospital- and regional-level variables in the sensitivity analyses in hospitals and regions with at least one ICU beds for Model 3.**

| **Variables** | **Odds ratio (95% CI)** | **IOR-80%** | **POOR** |
| --- | --- | --- | --- |
| **Hospital-level** |  |  |  |
| Hospitals with ICU beds | Omitted | Omitted | Omitted |
| ICU beds | 1.09 (1.07, 1.12) | (0.24, 5.00) | 47.0 |
| HCU beds | 0.99 (0.98, 1.00) | (0.22, 4.53) | 49.8 |
| Acute-care beds | 1.00 (1.00, 1.00) | (0.22, 4.56) | 50.0 |
| Academic hospital | 1.09 (0.68, 1.74) | (0.24, 4.97) | 47.1 |
| Tertiary emergency hospital | 0.56 (0.41, 0.76) | (0.12, 2.55) | 31.1 |
| Ambulance cases | 1.00 (1.00, 1.00) | (0.22, 4.57) | 50.0 |
| IMV cases | 1.00 (1.00, 1.00) | (0.22, 4.56) | 50.0 |
| **Regional-level** |  |  |  |
| Regions with ICU beds | Omitted | Omitted | Omitted |
| ICU beds per million | 1.12 (1.07, 1.18) | (0.21, 5.97) | 46.4 |
| HDU beds per million | 1.01 (0.99, 1.04) | (0.19, 5.38) | 49.6 |
| Acute-care beds per million | 1.00 (1.00, 1.00) | (0.19, 5.31) | 50.0 |

Model 3: Multilevel logistic regression with patient- and cluster-level variables and random intercepts for clusters.

CI, confidence interval; IOR, interval odds ratio; POOR, proportion of opposed odds ratios; ICU, intensive care unit; HDU, high-dependency care unit; IMV, invasive mechanical ventilation.

**Supplemental Table 11. Results of multilevel logistic regression analyses of Models 2 and 3 with hospital clusters in the sensitivity analysis of hospitals with at least one ICU beds.**

|  | **Model 2** |  | **Model 3** |  |
| --- | --- | --- | --- | --- |
|  | **Odds ratio** |  | **Odds ratio** |  |
| **Variables** | **(95% CI)** | **P** | **(95% CI)** | **P** |
| **Patient-level** |  |  |  |  |
| Age | 0.99 (0.99, 1.00) | <0.001 | 0.99 (0.99, 1.00) | <0.001 |
| Male | 0.96 (0.92, 1.00) | 0.034 | 0.96 (0.92, 1.00) | 0.036 |
| BMI at admission |  |  |  |  |
| < 18.5 | 0.77 (0.72, 0.81) | <0.001 | 0.77 (0.72, 0.81) | <0.001 |
| 18.5–24.9 | Ref. |  | Ref. |  |
| 25.0–29.9 | 1.03 (0.98, 1.09) | 0.272 | 1.03 (0.98, 1.09) | 0.268 |
| ≥ 30.0 | 0.85 (0.77, 0.93) | <0.001 | 0.85 (0.77, 0.93) | <0.001 |
| Missing | 0.49 (0.46, 0.52) | <0.001 | 0.49 (0.46, 0.52) | <0.001 |
| CCI | 1.03 (1.01, 1.04) | <0.001 | 1.03 (1.01, 1.04) | <0.001 |
| Cognitive function |  |  |  |  |
| No dementia | Ref. |  | Ref. |  |
| Mild dementia | 0.89 (0.83, 0.95) | <0.001 | 0.89 (0.83, 0.95) | <0.001 |
| Moderate/severe dementia | 0.93 (0.86, 1.00) | 0.062 | 0.93 (0.86, 1.00) | 0.057 |
| Long-term care-needs |  |  |  |  |
| No care-needs | Ref. |  | Ref. |  |
| SL1-2 & CNL1-2 | 1.01 (0.45, 2.27) | 0.979 | 1.01 (0.45, 2.26) | 0.988 |
| CNL3-5 | 2.05 (0.86, 4.89) | 0.106 | 2.03 (0.85, 4.84) | 0.111 |
| Home medical care | 0.48 (0.44, 0.52) | <0.001 | 0.48 (0.44, 0.52) | <0.001 |
| Weekend admission | 1.15 (1.10, 1.20) | <0.001 | 1.15 (1.10, 1.20) | <0.001 |
| Location before admission |  |  |  |  |
| Home | Ref. |  | Ref. |  |
| Other hospitals | 1.13 (1.05, 1.21) | 0.001 | 1.13 (1.05, 1.21) | 0.001 |
| Nursing home | 0.74 (0.67, 0.81) | <0.001 | 0.74 (0.67, 0.81) | <0.001 |
| Ambulance use | 1.36 (1.29, 1.44) | <0.001 | 1.36 (1.29, 1.44) | <0.001 |
| Emergency admission | 0.63 (0.59, 0.67) | <0.001 | 0.63 (0.59, 0.67) | <0.001 |
| Surgery | 2.75 (2.57, 2.94) | <0.001 | 2.75 (2.57, 2.94) | <0.001 |
| CPR | 0.43 (0.41, 0.46) | <0.001 | 0.43 (0.40, 0.46) | <0.001 |
| Length of stay before IMV |  |  |  |  |
| On the day of admission | Ref. |  | Ref. |  |
| On the next day of admission | 1.71 (1.61, 1.82) | <0.001 | 1.71 (1.61, 1.82) | <0.001 |
| Day 3–6 | 1.70 (1.60, 1.81) | <0.001 | 1.70 (1.60, 1.81) | <0.001 |
| Day 7– | 1.41 (1.32, 1.50) | <0.001 | 1.40 (1.32, 1.50) | <0.001 |
| Primary diagnoses |  |  |  |  |
| Acute heart failure | 0.69 (0.65, 0.74) | <0.001 | 0.69 (0.65, 0.74) | <0.001 |
| Post cardiac arrest | 1.14 (1.06, 1.24) | 0.001 | 1.14 (1.06, 1.24) | 0.001 |
| Acute coronary syndrome | 0.78 (0.73, 0.84) | <0.001 | 0.78 (0.73, 0.84) | <0.001 |
| Stroke | 2.98 (2.77, 3.20) | <0.001 | 2.98 (2.77, 3.20) | <0.001 |
| Cancer | 1.13 (1.05, 1.22) | 0.001 | 1.14 (1.05, 1.23) | 0.001 |
| Aortic dissection or aneurysm | 3.17 (2.92, 3.45) | <0.001 | 3.17 (2.92, 3.45) | <0.001 |
| Abdominal diseases | 1.73 (1.60, 1.88) | <0.001 | 1.73 (1.60, 1.88) | <0.001 |
| Pneumonia | 0.56 (0.51, 0.61) | <0.001 | 0.56 (0.51, 0.61) | <0.001 |
| Trauma | 1.15 (1.06, 1.26) | 0.001 | 1.15 (1.06, 1.26) | 0.001 |
| Sepsis | 2.17 (1.96, 2.41) | <0.001 | 2.18 (1.97, 2.41) | <0.001 |
| Aspiration | 0.71 (0.64, 0.79) | <0.001 | 0.71 (0.64, 0.79) | <0.001 |
| Chronic respiratory diseases | 0.39 (0.33, 0.45) | <0.001 | 0.39 (0.33, 0.45) | <0.001 |
| Home to nearest ICU, km | 1.01 (1.00, 1.01) | <0.001 | 1.01 (1.00, 1.01) | <0.001 |
| **Hospital-level** |  |  |  |  |
| Hospitals with ICU beds |  |  | Omitted |  |
| ICU beds |  |  | 1.09 (1.07, 1.12) | <0.001 |
| HDU beds |  |  | 0.99 (0.98, 1.00) | 0.186 |
| Acute-care beds |  |  | 1.00 (1.00, 1.00) | 0.014 |
| Academic hospital |  |  | 1.09 (0.68, 1.74) | 0.722 |
| Tertiary emergency hospital |  |  | 0.56 (0.41, 0.76) | <0.001 |
| Ambulance cases |  |  | 1.00 (1.00, 1.00) | 0.497 |
| IMV cases |  |  | 1.00 (1.00, 1.00) | 0.221 |

Model 2: multilevel logistic regression with patient-level covariates and random intercepts for clusters. Model 3: multilevel logistic regression with patient-level variables, cluster-level variables, and random intercepts for clusters.

ICU, intensive care unit; HDU, high-dependency care unit; SMD, standardised mean difference; SD, standard deviation; BMI, body mass index; CCI, Charlson Comorbidity Index; SL, support level; CNL, care needs level; CPR, cardiopulmonary resuscitation; IMV, invasive mechanical ventilation; IQR, interquartile range.

**Supplemental Table 12. Results of multilevel logistic regression analyses of Models 2 and 3 with regional clusters in the sensitivity analysis of regions with at least one ICU beds.**

|  | **Model 2** |  | **Model 3** |  |
| --- | --- | --- | --- | --- |
|  | **Odds ratio** |  | **Odds ratio** |  |
| **Variables** | **(95% CI)** | **P** | **(95% CI)** | **P** |
| **Patient-level** |  |  |  |  |
| Age | 0.99 (0.99, 0.99) | <0.001 | 0.99 (0.99, 0.99) | <0.001 |
| Male | 0.96 (0.93, 0.99) | 0.020 | 0.96 (0.93, 0.99) | 0.020 |
| BMI at admission |  |  |  |  |
| < 18.5 | 0.74 (0.71, 0.78) | <0.001 | 0.74 (0.71, 0.78) | <0.001 |
| 18.5–24.9 | Ref. |  | Ref. |  |
| 25.0–29.9 | 1.04 (0.99, 1.09) | 0.132 | 1.04 (0.99, 1.09) | 0.132 |
| ≥30.0 | 0.88 (0.81, 0.96) | 0.003 | 0.88 (0.81, 0.96) | 0.003 |
| Missing | 0.50 (0.47, 0.53) | <0.001 | 0.50 (0.47, 0.53) | <0.001 |
| CCI | 1.03 (1.02, 1.04) | <0.001 | 1.03 (1.02, 1.04) | <0.001 |
| Cognitive function |  |  |  |  |
| No dementia | Ref. |  | Ref. |  |
| Mild dementia | 0.87 (0.82, 0.92) | <0.001 | 0.87 (0.82, 0.92) | <0.001 |
| Moderate/severe dementia | 0.79 (0.74, 0.84) | <0.001 | 0.79 (0.74, 0.84) | <0.001 |
| Long-term care-needs |  |  |  |  |
| No care-needs | Ref. |  | Ref. |  |
| SL1-2 & CNL1-2 | 0.37 (0.22, 0.62) | <0.001 | 0.37 (0.22, 0.63) | <0.001 |
| CNL3-5 | 0.27 (0.16, 0.46) | <0.001 | 0.27 (0.16, 0.46) | <0.001 |
| Home medical care | 0.46 (0.42, 0.49) | <0.001 | 0.46 (0.42, 0.50) | <0.001 |
| Weekend admission | 1.14 (1.10, 1.19) | <0.001 | 1.14 (1.10, 1.19) | <0.001 |
| Location before admission |  |  |  |  |
| Home | Ref. |  | Ref. |  |
| Other hospitals | 1.12 (1.05, 1.19) | <0.001 | 1.12 (1.05, 1.19) | <0.001 |
| Nursing home | 0.72 (0.66, 0.78) | <0.001 | 0.72 (0.66, 0.78) | <0.001 |
| Ambulance use | 1.57 (1.50, 1.65) | <0.001 | 1.57 (1.50, 1.65) | <0.001 |
| Emergency admission | 0.62 (0.58, 0.65) | <0.001 | 0.62 (0.58, 0.65) | <0.001 |
| Surgery | 2.71 (2.56, 2.86) | <0.001 | 2.71 (2.56, 2.86) | <0.001 |
| CPR | 0.39 (0.37, 0.42) | <0.001 | 0.39 (0.37, 0.42) | <0.001 |
| Length of stay before IMV |  |  |  |  |
| On the day of admission | Ref. |  | Ref. |  |
| On the next day of admission | 1.61 (1.53, 1.70) | <0.001 | 1.61 (1.53, 1.70) | <0.001 |
| Day 3–6 | 1.69 (1.60, 1.79) | <0.001 | 1.69 (1.60, 1.79) | <0.001 |
| Day 7– | 1.35 (1.28, 1.42) | <0.001 | 1.35 (1.28, 1.42) | <0.001 |
| Primary diagnoses |  |  |  |  |
| Acute heart failure | 0.73 (0.69, 0.77) | <0.001 | 0.73 (0.69, 0.77) | <0.001 |
| Post cardiac arrest | 1.45 (1.35, 1.56) | <0.001 | 1.45 (1.35, 1.56) | <0.001 |
| Acute coronary syndrome | 0.80 (0.75, 0.86) | <0.001 | 0.80 (0.75, 0.86) | <0.001 |
| Stroke | 2.69 (2.53, 2.87) | <0.001 | 2.69 (2.53, 2.87) | <0.001 |
| Cancer | 1.11 (1.04, 1.19) | 0.001 | 1.11 (1.04, 1.19) | 0.001 |
| Aortic dissection or aneurysm | 3.33 (3.10, 3.59) | <0.001 | 3.33 (3.10, 3.59) | <0.001 |
| Abdominal diseases | 1.39 (1.30, 1.49) | <0.001 | 1.39 (1.30, 1.49) | <0.001 |
| Pneumonia | 0.52 (0.48, 0.56) | <0.001 | 0.52 (0.48, 0.56) | <0.001 |
| Trauma | 1.20 (1.11, 1.30) | <0.001 | 1.20 (1.11, 1.30) | <0.001 |
| Sepsis | 2.10 (1.92, 2.29) | <0.001 | 2.10 (1.93, 2.29) | <0.001 |
| Aspiration | 0.73 (0.66, 0.80) | <0.001 | 0.73 (0.66, 0.80) | <0.001 |
| Chronic respiratory diseases | 0.36 (0.31, 0.41) | <0.001 | 0.36 (0.31, 0.41) | <0.001 |
| Home to nearest ICU, km | 1.00 (1.00, 1.00) | 0.003 | 1.00 (1.00, 1.00) | 0.003 |
| **Regional-level** |  |  |  |  |
| Regions with ICU beds |  |  | Omitted |  |
| ICU beds per million |  |  | 1.12 (1.07, 1.18) | <0.001 |
| HDU beds per million |  |  | 1.01 (0.99, 1.04) | 0.346 |
| Acute-care beds per million |  |  | 1.00 (1.00, 1.00) | 0.390 |

Model 2: multilevel logistic regression with patient-level covariates and random intercepts for clusters. Model 3: multilevel logistic regression with patient-level variables, cluster-level variables, and random intercepts for clusters.

ICU, intensive care unit; HDU, high-dependency care unit; SMD, standardised mean difference; SD, standard deviation; BMI, body mass index; CCI, Charlson Comorbidity Index; SL, support level; CNL, care needs level; CPR, cardiopulmonary resuscitation; IMV, invasive mechanical ventilation; IQR, interquartile range.
